# Handling Missing Data in Transmission Disequilibrium Test in Nuclear Families with One Affected Offspring

**DOI:** 10.1371/journal.pone.0046100

**Published:** 2012-10-08

**Authors:** Gulhan Bourget

**Affiliations:** Department of Mathematics, California State University, Fullerton, California, United States of America; University of California, Irvine, United States of America

## Abstract

The Transmission Disequilibrium Test (TDT) compares frequencies of transmission of two alleles from heterozygote parents to an affected offspring. This test requires all genotypes to be known from all members of the nuclear families. However, obtaining all genotypes in a study might not be possible for some families, in which case, a data set results in missing genotypes. There are many techniques of handling missing genotypes in parents but only a few in offspring. The robust TDT (rTDT) is one of the methods that handles missing genotypes for all members of nuclear families [with one affected offspring]. Even though all family members can be imputed, the rTDT is a conservative test with low power. We propose a new method, Mendelian Inheritance TDT (MITDT-ONE), that controls type I error and has high power. The MITDT-ONE uses Mendelian Inheritance properties, and takes population frequencies of the disease allele and marker allele into account in the rTDT method. One of the advantages of using the MITDT-ONE is that the MITDT-ONE can identify additional significant genes that are not found by the rTDT. We demonstrate the performances of both tests along with Sib-TDT (S-TDT) in Monte Carlo simulation studies. Moreover, we apply our method to the type 1 diabetes data from the Warren families in the United Kingdom to identify significant genes that are related to type 1 diabetes.

## Introduction

The Transmission Disequilibrium Test (TDT) is the most widely used family-based test for linkage disequilibrium [1], [2]. It was first introduced to handle one affected offspring in a nuclear family, and was later extended to two or more affected offspring, and to multi-allelic markers as well. The TDT is a test for linkage in the presence of linkage disequilibrium [1], [2].

The TDT compares frequencies of the transmission of two alleles from heterozygote parents to an affected offspring. The TDT requires complete genotypes from parents and offspring. However, sometimes genotypes may not be available. If genotypes of parents are missing, including only complete cases [3], [4], [5], [6], [7], or reconstructing missing parental genotypes by assuming a missing at random (MAR) model [8] have been suggested as common approaches in practice. However, if parental genotypes are missing due to his genotype at the locus of interest, then the informatively missing model is more appropriate than the MAR model [9]. Also, including only complete families and families with only one parent missing in informatively missing parent(s) [3], [6], [7], [10] reconstructing parental genotypes from their affected offspring [2], or from affected and unaffected siblings (Reconstruction-Combined TDT) [4], [11], or completely ignoring parental genotypes and comparing frequencies of genotypes of unaffected and affected offspring (S-TDT) [12], [13],[14], [15], or combining different data sets from families with parental genotypes and from families with missing parental genotype data but whose siblings' genotypes are unaffected (C-TDT) [12] has been also proposed as alternative approaches.

The robust TDT (rTDT) was proposed to handle any missing genotypes in a nuclear family with one affected offspring and bi-allelic marker [16]. The rTDT does not assume any missing model, and defines an interval estimate of TDT by considering all possible completions of missing genotypes. Sebastiani et al. [16] claimed that rTDT has more power than TDT. The simulation study was not performed, and the claim of having more power than TDT was shown mathematically for a specific missing pattern for each family [16]. That is, they assumed that missing families have the same form: the genotype of one parent is missing, the other parent has a heterozygous genotype, and the affected child has homozygous genotype [see Discussion section for more details]. This specific missing pattern for each family is not a reasonable assumption in practice. Alpargu (Bourget) [17] defined the rTDT for *two* affected offspring, and showed in simulation studies that rTDT was too conservative, and had low power. Because of its poor performance, the Mendelian Inheritance-Transmission Disequilibrium Test (MI-TDT), which takes population frequencies of the disease allele 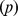 and marker allele 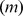 into account in rTDT, was proposed [17]. The MI-TDT performed better than rTDT by controlling type I error rates and having high power. Since, MI-TDT outperformed rTDT, in this paper we propose the Mendel Inheritance-Transmission Disequilibrium Test (MITDT-ONE) for *one* affected offspring. The MITDT-ONE considers 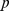 and 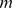 in rTDT. The simulation study replicating real life scenarios such as different missing models and different genetic models shows that MITDT-ONE outperforms rTDT by providing better control of type I error rates and producing higher power.

## Methods

We demonstrate the features of rTDT and MITDT-ONE with an example. We assume that we have genotypes of nuclear families with one affected offspring, and bi-allelic markers with alleles 1 and 2. In a given data set, there are (1,1), (1,2), or (2,2) complete genotypes or (0,0) missing genotypes. For each family, there are three genotypes with the first two genotypes for parents and the last genotype for offspring (e.g., (1,2)(1,1)(1,2)). If at least one of the genotypes is unknown, then the data is called incomplete. Otherwise it is called complete. Hence, a whole data set has two parts for a given marker: complete and incomplete trio genotypes.

The TDT considers transmission from heterozygote parents (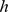) to affected offspring. Let 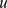 be the number of 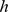 that transmit allele 1 to an affected offspring, and 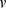 be the number of 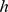 that transmit allele 2 to an affected offspring. Then, the TDT statistic for complete data
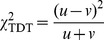
(1)tests linkage (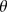) between a disease and a marker locus in the presence of linkage disequilibrium (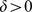 or 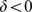) [1]. Under the null hypothesis of no linkage (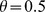), 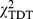 follows a central chi-square distribution with 1 degree of freedom (df).

We construct interval estimates of MITDT-ONE and rTDT as follows: (1) compute maximum and minimum increments in 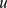 and 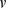 by considering all possible admissible completions of missing genotypes (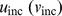 for maximum increments of 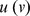), (2) find population frequencies of disease allele 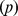, and marker allele (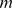), and finally, (3) compute maximum and minimum values of 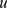 and 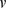 (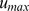 and 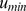 for 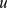 and 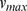 and 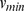 for 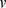). While all three steps are involved in MITDT-ONE, rTDT does not require step (2). This is the only important difference between two methods. However, MITDT-ONE requires the value of 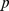, which is difficult to know in some diseases. We can overcome the knowledge of 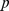 by assuming 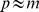 because McGinnis (1998) [18] showed that TDT is able to detect linkage, and its power exceeds 0.5 only when 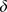 is close to its most positive value 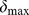 (see the definition of 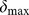 in the following section) when 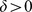, and allele frequencies 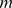 and 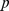 are similar in magnitude at marker and disease locus.

For complete families, let us assume that we have 50 heterozygote parents (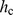) in which 35 of them transmit allele 1 (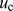), and 15 of them transmit allele 2 (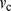). Using (1), we compute 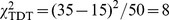. The chi-square distribution with 1 df at 5% nominal level is 3.84. Based on only complete cases, we reject the null hypothesis of no linkage at 5% nominal level. Now, assume 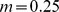 and 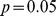 with two missing families as in Table 1.

**Table 1:** Two missing cases.

Family	Parents	Children
1	(0,0)(1,2)	(0,0)
2	(1,2)(1,1)	(0,0)

The first step of imputing missing cases involves only possible admissible completions. The MITDT-ONE and rTDT (as does TDT) consider families with at least one heterozygote parent. For example, if the incomplete case is (1,1)(0,0)(1,2), we do not consider the completion (1,1)(2,2)(1,2) because both parents have homozygous genotypes. Moreover, in family 2 above, (1,2)(1,1)(2,2) is not a possible admissible completion because the only possible completions for offspring are (1,1) or (1,2). All possible admissible genotypes are defined in Table 2.

**Table 2:** Admissible cases.

Family	Scenario	Parent	Children	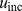	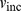
1	1	(1,1)(1,2)	(1,1)	1	0
	2	(1,1)(1,2)	(1,2)	0	1
	3	(2,2)(1,2)	(1,2)	1	0
	4	(2,2)(1,2)	(2,2)	0	1
	5	(1,2)(1,2)	(1,1)	2	0
	6	(1,2)(1,2)	(1,2)	1	1
	7	(1,2)(1,2)	(2,2)	0	2
2	8	(1,2)(1,1)	(1,1)	1	0
	9	(1,2)(1,1)	(1,2)	0	1

Under the null hypothesis 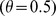, heterozygote parent transmits allele 1 but not allele 2 to an affected offspring with probability 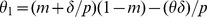, and the same parent transmits allele 2 but not allele 1 to an affected offspring with probability 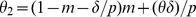, where 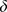 is the coefficient of disequilibrium, 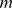 is the frequency of the marker allele 1, and 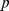 is the population relative frequency of disease allele [19]. The 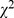 statistic compares the number of transmissions with probabilities 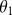 and 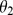. It can be shown that these probabilities are the same under the null hypothesis. Thus, the expected number of transmissions are the same. Thus, 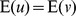. However, the probabilities are different when there is linkage, and hence the number of transmissions are different. This means that the 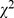 statistic is related to the parameters 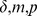, and 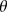.

All these families have equal probabilities of being considered under the null hypothesis of no linkage. However, MITDT-ONE and rTDT consider increments in 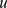 (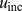) and 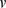 (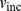). The exact maximum and minimum values of TDT in (1) are attained by rTDT. The interval estimate of rTDT is 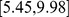. While the minimum value is attained when 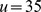 and 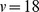 (scenarios 7 and 9), the maximum value is attained when 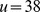 and 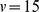 (scenarios 5 and 8). The interval estimate of MITDT-ONE is 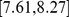 with the same completion of the families as rTDT.

Both tests use the same admissible cases and consider lower limits to identify significant genes. Both methods reject the null hypothesis of no linkage at 5% nominal level in the above example. The interval estimate of MITDT-ONE is always contained in the interval estimate of rTDT (see in Construction of the MITDT-ONE and rTDT for more details). It is important to note that MITDT-ONE and rTDT have the same minimum values for 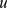 and 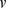 but differ at maximum values of 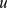 and 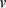. Therefore, MITDT-ONE will never have less power than rTDT. Since the MITDT-ONE has more power and controls type I error rates better, we suggest using the MITDT-ONE test instead of rTDT test.

### Construction of the MITDT-ONE and rTDT

There are 17 admissible missing cases in a nuclear family with one affected offspring (Table 3). Sebastiani et al. [16] proposed an interval estimate of rTDT for one affected offspring. They proceeded in the following way: 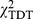 in (1) is a monotone convex function on a closed domain. Thus, it achieves its maximum and minimum values at one of its extreme points. The maximum and minimum values of 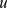 and 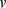 were considered to define the maximum and minimum values of 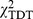. First, all possible admissible completions were identified (Tables 4 and 5), and then the maximum and minimum increments in 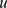 and 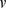 (Table 6) were defined as
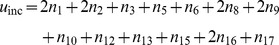


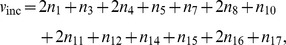
where 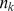 (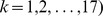 is the number of missing families in case 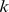. The maximum and minimum values of 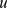 and 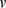 were defined as
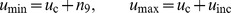


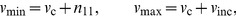
(2)where 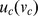 is the number of 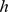 that transmit allele 1 (2) to affected offspring in complete data set. And finally, the interval estimate 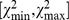 of rTDT was defined as

1. If 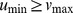, then
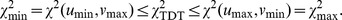

2. If 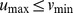, then
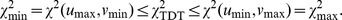

3. In all other cases:
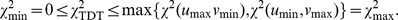

The value of 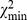 (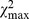) makes a decision against (conforming) the null hypothesis. If 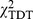 for complete data (i.e., missing data are ignored) and 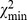 reach the conclusion of the alternative hypothesis (i.e., significant genes), and 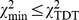, then rTDT affirms significant genes of complete data. Similarly, the value of 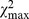 ratifies the insignificant genes if 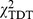 and 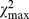 cannot reject the null hypothesis, and 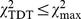. In all other scenarios, rTDT cannot verify any conclusions of complete data.

**Table 3:** Number of missing cases in a family with one affected offspring.

	Offspring Genotype					
Case	Parental Genotype	(0,0)	(1,1)	(1,2)	(2,2)	Total
1	(0,0) (0,0)	+	+	+	+	4
2	(0,0) (1,1)	+	+	+	−	3
3	(0,0) (1,2)	+	+	+	+	4
4	(0,0) (2,2)	+	−	+	+	3
5	(1,1) (1,2)	+	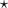	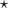	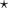	1
6	(1,2) (1,2)	+	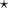	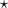	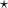	1
7	(1,2) (2,2)	+	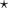	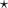	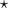	1
Total number of admissible incomplete trios						17

The symbols 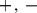, and 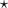 denote possible incomplete, impossible incomplete, and complete cases, respectively.

**Table 4:** List of admissible completions for cases 1–8.

Case	Incomplete Genotypes		Admissible Completions		Increments	
k	Parents	Offspring	Parents	Offspring	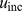	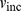
1	(0, 0)(0, 0)	(0, 0)	(1,1) (1,1)	(1,1)	0	0
			(1,1) (1,2)	(1,1)	1	0
			(1,1) (1,2)	(1,2)	0	1
			(1,1) (2,2)	(1,2)	0	0
			(1,2) (1,2)	(1,1)	2	0
			(1,2) (1,2)	(1,2)	1	1
			(1,2) (1,2)	(2,2)	0	2
			(2,2) (1,2)	(1,2)	1	0
			(2,2) (1,2)	(2,2)	0	1
			(2,2) (2,2)	(2,2)	0	0
2	(0, 0)(0, 0)	(1, 1)	(1,1) (1,1)	(1,1)	0	0
			(1,1) (1,2)	(1,1)	1	0
			(1,2) (1,2)	(1,1)	2	0
3	(0, 0)(0, 0)	(1, 2)	(1,1) (1,2)	(1,2)	0	1
			(1,1) (2,2)	(1,2)	0	0
			(1,2) (1,2)	(1,2)	1	1
			(1,2) (2,2)	(1,2)	1	0
4	(0, 0)(0, 0)	(2, 2)	(1,2) (1,2)	(2,2)	0	2
			(1,2) (2,2)	(2,2)	0	1
			(2,2) (2,2)	(2,2)	0	0
5	(0, 0)(1, 1)	(0, 0)	(1,1) (1,1)	(1,1)	0	0
			(1,2) (1,1)	(1,1)	1	0
			(1,2) (1,1)	(1,2)	0	1
			(2,2) (1,1)	(1,2)	0	0
6	(0, 0)(1, 1)	(1, 1)	(1,1) (1,1)	(1,1)	0	0
			(1,2) (1,1)	(1,1)	1	0
7	(0, 0)(1, 1)	(1, 2)	(1,2) (1,1)	(1,2)	0	1
			(2,2) (1,1)	(1,2)	0	0
8	(0.0) (1,2)	(0,0)	(1,1) (1,2)	(1,1)	1	0
			(1,1) (1,2)	(1,2)	0	1
			(1,2) (1,2)	(1,1)	2	0
			(1,2) (1,2)	(1,2)	1	1
			(1,2) (1,2)	(2,2)	0	2
			(2,2) (1,2)	(1,2)	1	0
			(2,2) (1,2)	(2,2)	0	1

**Table 5:** List of admissible completions for cases 9–17.

Case	Incomplete Genotypes		Admissible Completions		Increments	
k	Parents	Offspring	Parents	Offspring	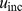	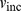
9	(0, 0)(1, 2)	(1, 1)	(1,1) (1,2)	(1,1)	1	0
			(1,2) (1,2)	(1,1)	2	0
10	(0, 0)(1, 2)	(1, 2)	(1,1) (1,2)	(1,2)	0	1
			(1,2) (1,2)	(1,2)	1	1
			(2,2) (1,2)	(1,2)	1	0
11	(0, 0)(1, 2)	(2, 2)	(1,2) (1,2)	(2,2)	0	2
			(2,2) (1,2)	(2,2)	0	1
12	(0, 0)(2, 2)	(0, 0)	(1,1) (2,2)	(1,2)	0	0
			(1,2) (2,2)	(1,2)	1	0
			(1,2) (2,2)	(2,2)	0	1
			(2,2) (2,2)	(2,2)	0	0
13	(0, 0)(2, 2)	(1, 2)	(1,1) (2,2)	(1,2)	0	0
			(1,2) (2,2)	(1,2)	1	0
			(2,2) (2,2)	(1,2)	0	0
14	(0, 0)(2, 2)	(2, 2)	(1,2) (2,2)	(2,2)	0	1
			(2,2) (2,2)	(2,2)	0	0
15	(1, 1)(1, 2)	(0, 0)	(1,1) (1,2)	(1,1)	1	0
			(1,1) (1,2)	(1,2)	0	1
16	(1, 2)(1, 2)	(0, 0)	(1,2) (1,2)	(1,1)	2	0
			(1,2) (1,2)	(1,2)	1	1
			(1,2) (1,2)	(2,2)	0	2
17	(1, 2)(2, 2)	(0, 0)	(1,2) (2,2)	(1,2)	1	0
			(1,2) (2,2)	(2,2)	0	1

**Table 6:** Admissible increments of 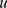 and 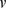.

Case	Parents	Offspring	Increment 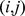						Min.	Max.
			(0,0)	(1,0)	(2,0)	(1,1)	(0,1)	(0,2)	Inc	Inc
1	(0,0) (0,0)	(0,0)	+	+	+	+	+	+	0	2
2		(1,1)	+	+	+	−	−	−	0	2
3		(1,2)	+	+	−	+	+	−	0	2
4		(2,2)	+	−	−	−	+	+	0	2
5	(0,0) (1,1)	(0,0)	+	+	−	−	+	−	0	1
6		(1,1)	+	+	−	−	−	−	0	1
7		(1,2)	+	−	−	−	+	−	0	1
8	(0,0) (1,2)	(0,0)	−	+	+	+	+	+	1	2
9		(1,1)	−	+	+	−	−	−	1	2
10		(1,2)	−	+	−	+	+	−	1	2
11		(2,2)	−	−	−	−	+	+	1	2
12	(0,0) (2,2)	(0,0)	+	+	−	−	+	−	0	1
13		(1,2)	+	+	−	−	−	−	0	1
14		(2,2)	+	−	−	−	+	−	0	1
15	(1,1) (1,2)	(0,0)	−	+	−	−	+	−	1	1
16	(1,2) (1,2)	(0,0)	−	−	+	+	−	+	2	2
17	(1,2) (2,2)	(0,0)	−	+	−	−	+	−	1	1

In 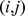, 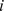 and 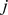 represent the increments in 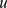 and 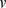, respectively. The plus (minus) sign indicates that the increment is plausible (not plausible). The last two columns show the maximum and minimum increments in each cases.

Sebastiani et al. [16] did not run any simulation study to demonstrate the performance of rTDT. They theoretically showed that if all missing families are in case 9, which is not a reasonable assumption in practice, then rTDT has higher power than the classical 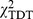. Since the power of TDT depends on linkage disequilibrium 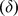, and relative frequencies of marker allele (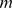) and disease allele (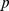) [20], we ran simulation studies to take into account different realistic disease models and missing models, involving 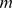 and 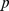. The simulation results show that rTDT overestimates the values of 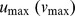 (results are not shown), and hence becomes a conservative test with low power. Since 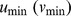 does not involve 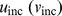, we decided to scale down 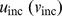 to have a smaller value of 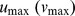 for MITDT-ONE. One way to achieve this goal is to involve 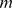 and 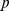 in scaling. These parameters appear together in maximum linkage disequilibrium 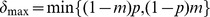 when linkage disequilibrium is positive 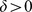, and 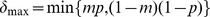 when linkage disequilibrium is negative 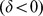 [18]. We scale 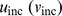 with 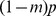 and 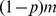 when 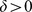, and define 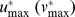 for MITDT-ONE as the average of these values. That is,
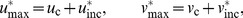
(3)where
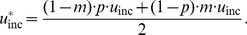
(4)Similarly, we can define 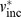 by replacing in (4) 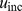 with 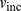.

Since TDT provides better power when linkage disequilibrium is at its maximum (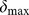) for 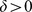, and 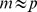 [18], we can reformulate (4) for real sample data as
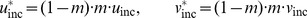
(5)


The lowest values of the interval estimates of rTDT and MITDT-ONE find significant genes when they are actually not. The way the interval estimate for MITDT-ONE constructed guarantees that its lowest interval estimate 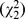 is always larger than the lowest interval estimate of rTDT 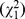. This fact can be shown theoretically in the following way: let us assume 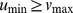 (the other two conditions in (31) can be shown similarly). Since 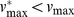, we have
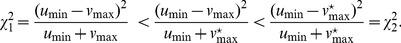
(6)


We claimed that rTDT is a conservative test. We have observed this through simulation study but not theoretically. The reason rTDT becomes conservative is that the value of 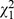, in general, falls below the value of chi-square distribution with 1 df at 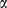 nominal level (for example, when 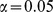, this value is 3.84).

## Results

### Simulation

We replicated the simulation study in [17] for one affected offspring. Let us assume a bi-allelic marker with alleles 1 and 2 which is linked to a bi-allelic disease locus with disease-predisposing allele 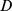 and non-predisposing allele 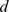. The penetrance for 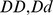 and 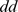 genotypes are 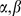 and 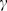, respectively, with 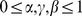, and the population frequencies for the marker with disease locus haplotype for 1D, 1d, 2D and 2d are 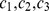 and 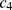, respectively, where 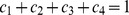. The population relative frequency of disease allele D is 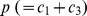. The frequencies of the marker alleles 1 and 2 are 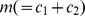 and 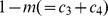, respectively. The recombination fraction between the disease and marker locus is 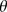, and the coefficient of disequilibrium is 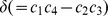. The probability of a heterozygote parent transmitting marker allele 1 to a particular affected child [18] is defined as
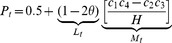
(7)

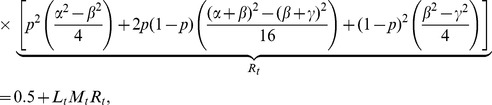
(8)where
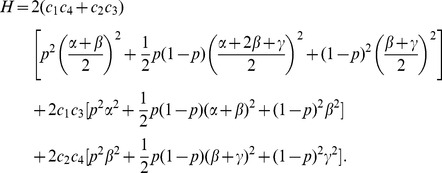



Our simulation study demonstrates realistic complex disease models. We generated 5,000 data sets for four different missing models and three genetics models (additive, dominant and recessive). In each simulation, we generated 100 families and each family consisted of one affected and one unaffected offspring, and 50 heterozygote fathers and 50 heterozygote mothers. In disease models, the probabilities of an affected child given the homozygosity (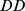), heterozygosity (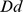), and absence of the disease alleles (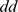) are defined as 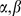, and 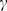, respectively. The values of these parameters were as 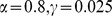 for dominant 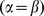, additive (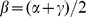), and recessive models 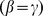. In missing models, we consider (1) Missing Completely at Random (MCAR) for all genotypes, (2) informative missing for parental genotypes and MCAR for offspring genotypes, (3) informative missing for all genotypes, and (4) MCAR for parental genotypes and informative missing for offspring genotypes. A model is called “informatively missing” if at least two of the 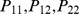 are not equal, where 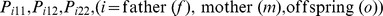 are missing rates for f, m, and o with (1,1), (1,2) and (2,2) genotypes, respectively. In Table 7, the first column 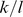 denotes the missing patterns (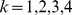) and missing rates (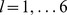).

**Table 7:** Missing model (MM) and missing rates (MR).

	Missing Rates		
MM/MR	Father	Mother	Offspring
	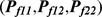	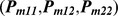	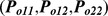
1/1	(0.10,0.10,0.10)	(0.10,0.10,0.10)	(0.10,0.10,0.10)
2/1	(0.05, 0.05, 0.10)	(0.05, 0.05, 0.10)	(0.10,0.10,0.10)
2/2	(0.05, 0.075, 0.10)	(0.05, 0.075, 0.10)	(0.10,0.10,0.10)
2/3	(0.05, 0.10, 0.10)	(0.05, 0.10, 0.10)	(0.10,0.10,0.10)
2/4	(0.10, 0.05, 0.05)	(0.10, 0.05, 0.05)	(0.10,0.10,0.10)
2/5	(0.10, 0.075, 0.05)	(0.10, 0.075, 0.05)	(0.10,0.10,0.10)
2/6	(0.10, 0.10, 0.05)	(0.10, 0.10, 0.05)	(0.10,0.10,0.10)
3/1	(0.05, 0.05, 0.10)	(0.05, 0.05, 0.10)	(0.05, 0.05, 0.10)
3/2	(0.05, 0.075, 0.10)	(0.05, 0.075, 0.10)	(0.05, 0.075, 0.10)
3/3	(0.05, 0.10, 0.10)	(0.05, 0.10, 0.10)	(0.05, 0.10, 0.10)
3/4	(0.10, 0.05, 0.05)	(0.10, 0.05, 0.05)	(0.10, 0.05, 0.05)
3/5	(0.10, 0.075, 0.05)	(0.10, 0.075, 0.05)	(0.10, 0.075, 0.05)
3/6	(0.10, 0.10, 0.05)	(0.10, 0.10, 0.05)	(0.10, 0.10,0.05)
4/1	(0.10, 0.10, 0.10)	(0.10, 0.10, 0.10)	(0.05, 0.05, 0.10)
4/2	(0.10, 0.10, 0.10)	(0.10, 0.10, 0.10)	(0.05, 0.075, 0.10)
4/3	(0.10, 0.10, 0.10)	(0.10, 0.10, 0.10)	(0.05, 0.10, 0.10)
4/4	((0.10, 0.10, 0.10)	(0.10, 0.10, 0.10)	(0.10, 0.05, 0.05)
4/5	(0.10, 0.10, 0.10)	(0.10, 0.10, 0.10)	(0.10, 0.075, 0.05)
4/6	(0.10, 0.10, 0.10)	(0.10, 0.10, 0.10)	(0.10, 0.10,0.05)

Missing models:(1) Missing Completely at Random (MCAR) for all genotypes, (2) informative missing for parental genotypes and MCAR for offspring genotypes, (3) informative missing for all genotypes, and (4) MCAR for parental genotypes and informative missing for offspring genotypes. 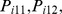 and 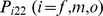 denote missing rates for father (f), mother (m), and offspring (o) with (1,1), (1,2), and (2,2) genotypes, respectively.

The performances of the methods were demonstrated by validity and power analysis. The S-TDT, which ignores genotypes of the parents and compares frequencies of the affected and unaffected offspring [see 14 for the computation of S-TDT], was included to compare our methods with one of the widely used family based methods. Since S-TDT completely ignores parental genotypes and requires unaffected offspring genotypes from these families, and also assumes affected offspring genotypes are available, none of the missing mechanism models were taken into account. It means that the type I error rates for S-TDT are all the same whatever the missing mechanism models are for a given 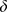 value.

In validity and power analysis tables, the TDT ignores missing cases and considers only complete cases, S-TDT ignores parental genotypes and considers only genotypes of affected and unaffected offspring of all 100 families (genotypes are all known), and MITDT and rTDT use all 100 families after construction of all possible admissible genotypes.

The most positive value of linkage disequilibrium is defined as 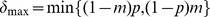 when 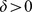, and the most negative value of linkage disequilibrium is defined as 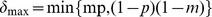 when 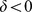. Since type I error rate and power results for 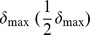 when 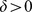 at 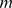 are equal to type I error rate and power results for 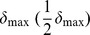 when 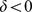 at 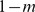, we only consider the values of 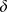 when 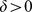. In the presence of positive linkage disequilibrium (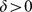), the null hypotheses are there is no linkage 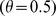 in validity analysis, and there is a complete linkage 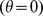 in power analysis. The values of 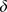 were chosen as moderate 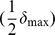 and maximum 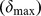 with 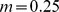 and 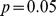.

### Validity Analysis

When 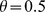, the probability that an informative parent transmits marker allele 1 to a particular affected child (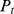) becomes 0.5 because 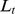 is zero in (8). That is, the value of 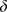 in 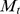 and the disease model in 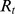 are not involved in validity analysis. It means that type I error rates are the same for every disease model.

All testing procedures (TDT, MITDT-ONE, rTDT) except S-TDT were valid tests at 1% and 5% significance levels (Tables 8 and 9). Since TDT, MITDT-ONE and rTDT takes also information about genotypes of parents into account as opposed to S-TDT, this information had a positive impact on the sizes of the tests. Since S-TDT had inflated type I errors, we excluded its performance in power analysis. Overall, MITDT-ONE outperformed rTDT by providing type I error rates close to the corresponding significance levels. The rTDT was the conservative test. Actually, this was the main reason for us to propose a new test that controls type I error rates better. The results in Tables 8 and 9 show that the MITDT-ONE achieved this goal. Since MITDT-ONE (and rTDT) does not assume any specific missing models, we suggest that MITDT-ONE should be preferred over some widely used family based testing procedures.

**Table 8:** Type I error rates at 1% significance level under the null hypothesis of 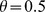.

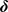	GM	MM/MR	S-TDT	TDT	rTDT	MI-TDT
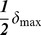	D, A, R	1/1	0.024	0.009	0	0.007
		2/1	0.024	0.009	0	0.007
		2/2		0.01	0	0.007
		2/3		0.009	0	0.007
		2/4		0.009	0	0.006
		2/5		0.009	0	0.007
		2/6		0.009	0	0.007
		3/1	0.024	0.01	0	0.007
		3/2		0.011	0	0.007
		3/3		0.01	0	0.007
		3/4		0.009	0	0.007
		3/5		0.01	0	0.008
		3/6		0.01	0	0.009
		4/1	0.024	0.01	0	0.007
		4/2		0.01	0	0.007
		4/3		0.01	0	0.007
		4/4		0.01	0	0.009
		4/5		0.01	0	0.009
		4/6		0.01	0	0.009
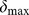	D, A, R	1/1	0.022	0.007	0	0.006
		2/1	0.022	0.007	0	0.004
		2/2		0.007	0	0.005
		2/3		0.007	0	0.006
		2/4		0.008	0	0.004
		2/5		0.008	0	0.004
		2/6		0.008	0	0.005
		3/1	0.022	0.009	0	0.005
		3/2		0.009	0	0.005
		3/3		0.008	0	0.006
		3/4		0.008	0	0.005
		3/5		0.009	0	0.006
		3/6		0.008	0	0.007
		4/1	0.022	0.008	0	0.006
		4/2		0.008	0	0.006
		4/3		0.008	0	0.006
		4/4		0.008	0	0.007
		4/5		0.008	0	0.007
		4/6		0.008	0	0.007

**Table 9:** Type I error rates at 5% significance level under the null hypothesis of 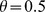.

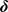	GM	MM/MR	S-TDT	TDT	rTDT	MI-TDT
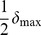	D, A, R	1/1	0.104	0.05	0	0.04
		2/1	0.104	0.048	0	0.036
		2/2		0.048	0	0.037
		2/3		0.05	0	0.04
		2/4		0.047	0.001	0.034
		2/5		0.047	0.001	0.034
		2/6		0.05	0.001	0.037
		3/1	0.104	0.052	0.001	0.035
		3/2		0.052	0	0.036
		3/3		0.052	0	0.041
		3/4		0.048	0.003	0.036
		3/5		0.053	0.002	0.04
		3/6		0.053	0.001	0.042
		4/1	0.104	0.053	0.001	0.039
		4/2		0.052	0	0.04
		4/3		0.052	0	0.041
		4/4		0.053	0.001	0.042
		4/5		0.053	0	0.043
		4/6		0.053	0	0.045
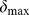	D, A, R	1/1	0.102	0.045	0	0.036
		2/1	0.102	0.043	0	0.031
		2/2		0.043	0	0.034
		2/3		0.045	0	0.036
		2/4		0.042	0.001	0.028
		2/5		0.042	0.001	0.03
		2/6		0.045	0.001	0.034
		3/1	0.102	0.045	0	0.032
		3/2		0.046	0	0.034
		3/3		0.047	0	0.037
		3/4		0.041	0.003	0.033
		3/5		0.047	0.002	0.037
		3/6		0.045	0.001	0.038
		4/1	0.102	0.046	0	0.036
		4/2		0.046	0	0.037
		4/3		0.047	0	0.037
		4/4		0.045	0	0.038
		4/5		0.046	0	0.039
		4/6		0.047	0	0.039

In column 2, D, A, and R represent dominant, additive, and recessive genetic models (GM), respectively.

### Power Analysis

In power analysis, the null hypothesis is that there is a complete linkage (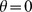). When 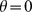, the probability of an informative parent transmitting marker allele 1 to a particular affected child (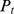) becomes greater than or equal to 0.5 because 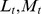, and 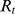 contribute to the value of 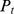. It means information from linkage disequilibrium and 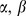 and 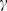 (parameters of disease model) have positive effect on power. This theoretical fact was also observed through simulation studies in Tables 10, 11, 12, 13, 14, 15. The pattern of power for all disease models, missing rates, missing models, and strength of linkage disequilibrium were the same for different significance levels (1% and 5%). However, the power values were better at 5% significance level than at 1% significance level.

**Table 10:** Power values at 1% significance level when alternative hypothesis is 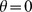.

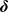	GM	MM/MR	S-TDT	TDT	rTDT	MI-TDT
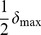	Dominant	1/1	0.291	0.769	0.072	0.829
		2/1	0.291	0.782	0.102	0.833
		2/2		0.785	0.098	0.835
		2/3		0.769	0.072	0.829
		2/4		0.78	0.18	0.815
		2/5		0.782	0.175	0.819
		2/6		0.767	0.145	0.823
		3/1	0.291	0.764	0.136	0.815
		3/2		0.776	0.111	0.826
		3/3		0.769	0.072	0.829
		3/4		0.795	0.449	0.83
		3/5		0.812	0.385	0.838
		3/6		0.778	0.294	0.833
		4/1	0.291	0.761	0.094	0.821
		4/2		0.762	0.08	0.823
		4/3		0.769	0.072	0.829
		4/4		0.769	0.298	0.826
		4/5		0.77	0.266	0.829
		4/6		0.777	0.244	0.835
	Additive	1/1	0.257	0.72	0.053	0.788
		2/1	0.257	0.729	0.077	0.792
		2/2		0.735	0.074	0.795
		2/3		0.72	0.053	0.788
		2/4		0.724	0.143	0.772
		2/5		0.731	0.139	0.774
		2/6		0.716	0.113	0.781
		3/1	0.257	0.714	0.105	0.77
		3/2		0.726	0.083	0.786
		3/3		0.72	0.053	0.788
		3/4		0.751	0.383	0.788
		3/5		0.767	0.327	0.795
		3/6		0.731	0.248	0.793
		4/1	0.257	0.713	0.068	0.778
		4/2		0.715	0.059	0.781
		4/3		0.72	0.053	0.788
		4/4		0.721	0.253	0.785
		4/5		0.724	0.218	0.788
		4/6		0.729	0.197	0.795

**Table 11:** Power analysis continues.

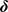	GM	MM/MR	S-TDT	TDT	rTDT	MI-TDT
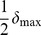	Recessive	1/1	0.231	0.68	0.042	0.756
		2/1	0.231	0.688	0.064	0.755
		2/2		0.693	0.061	0.761
		2/2		0.68	0.042	0.756
		2/2		0.686	0.119	0.734
		2/2		0.69	0.117	0.737
		2/2		0.676	0.097	0.746
		3/1	0.231	0.671	0.089	0.731
		3/2		0.682	0.068	0.748
		3/3		0.68	0.042	0.756
		¾		0.714	0.342	0.753
		3/5		0.728	0.287	0.76
		3/6		0.693	0.207	0.761
		4/1	0.231	0.673	0.055	0.744
		4/2		0.676	0.048	0.747
		4/3		0.68	0.042	0.756
		4/4		0.683	0.218	0.753
		4/5		0.686	0.186	0.756
		4/6		0.69	0.167	0.764
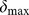	Dominant	1/1	0.021	0.009	0	0.007
		2/1	0.021	0.009	0	0.006
		2/2		0.009	0	0.006
		2/3		0.009	0	0.007
		2/4		0.009	0	0.005
		2/5		0.009	0	0.005
		2/6		0.009	0	0.007
		3/1	0.021	0.01	0	0.007
		3/2		0.01	0	0.007
		3/3		0.009	0	0.007
		¾		0.009	0	0.007
		3/5		0.01	0	0.007
		3/6		0.009	0	0.007
		4/1	0.021	0.009	0	0.007
		4/2		0.009	0	0.007
		4/3		0.009	0	0.007
		4/4		0.01	0	0.008
		4/5		0.009	0	0.008
		4/6		0.009	0	0.008

**Table 12:** Power analysis continues.

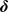	GM	MM/MR	S-TDT	TDT	rTDT	MI-TDT
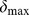	Additive	1/1	0.015	0.012	0	0.016
		2/1	0.015	0.009	0	0.014
		2/2		0.01	0	0.014
		2/3		0.012	0	0.016
		2/4		0.01	0	0.011
		2/5		0.01	0	0.011
		2/6		0.012	0	0.014
		3/1	0.015	0.009	0	0.012
		3/2		0.01	0	0.013
		3/3		0.012	0	0.016
		¾		0.011	0.001	0.014
		3/5		0.013	0	0.015
		3/6		0.013	0	0.016
		4/1	0.015	0.012	0	0.015
		4/2		0.012	0	0.015
		4/3		0.012	0	0.016
		4/4		0.012	0	0.017
		4/5		0.012	0	0.018
		4/6		0.013	0	0.018
	Recessive	1/1	0.014	0.014	0	0.021
		2/1	0.014	0.012	0	0.017
		2/2		0.013	0	0.019
		2/3		0.014	0	0.021
		2/4		0.013	0	0.015
		2/5		0.013	0	0.015
		2/6		0.013	0	0.018
		3/1	0.014	0.012	0	0.014
		3/2		0.013	0	0.017
		3/3		0.014	0	0.021
		¾		0.013	0.001	0.017
		3/5		0.016	0.001	0.019
		3/6		0.015	0	0.021
		4/1	0.014	0.012	0	0.02
		4/2		0.012	0	0.02
		4/3		0.014	0	0.021
		4/4		0.014	0	0.022
		4/5		0.014	0	0.023
		4/6		0.015	0	0.023

**Table 13:** Power values at 5% significance level when alternative hypothesis is 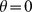.

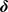	GM	MM/MR	S-TDT	TDT	rTDT	MI-TDT
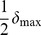	Dominant	1/1	0.571	0.911	0.249	0.941
		2/1	0.571	0.919	0.295	0.942
		2/2		0.919	0.287	0.943
		2/3		0.911	0.249	0.941
		2/4		0.919	0.484	0.938
		2/5		0.92	0.469	0.938
		2/6		0.913	0.402	0.937
		3/1	0.571	0.912	0.366	0.935
		3/2		0.916	0.314	0.939
		3/3		0.911	0.249	0.941
		¾		0.928	0.734	0.941
		3/5		0.933	0.67	0.944
		3/6		0.916	0.553	0.941
		4/1	0.571	0.909	0.309	0.936
		4/2		0.911	0.274	0.937
		4/3		0.911	0.249	0.941
		4/4		0.911	0.57	0.939
		4/5		0.913	0.535	0.94
		4/6		0.914	0.507	0.944
	Additive	1/1	0.524	0.885	0.198	0.92
		2/1	0.524	0.894	0.243	0.92
		2/2		0.893	0.234	0.922
		2/3		0.885	0.198	0.92
		2/4		0.894	0.421	0.914
		2/5		0.894	0.407	0.916
		2/6		0.885	0.343	0.914
		3/1	0.524	0.883	0.313	0.91
		3/2		0.89	0.262	0.916
		3/3		0.885	0.198	0.92
		3/4		0.904	0.681	0.918
		3/5		0.909	0.608	0.924
		3/6		0.89	0.489	0.918
		4/1	0.524	0.881	0.254	0.914
		4/2		0.883	0.221	0.916
		4/3		0.885	0.198	0.92
		4/4		0.885	0.504	0.917
		4/5		0.886	0.468	0.919
		4/6		0.888	0.44	0.923

**Table 14:** Power analysis continues.

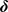	GM	MM/MR	S-TDT	TDT	rTDT	MI-TDT
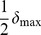	Recessive	1/1	0.498	0.862	0.168	0.905
		2/1	0.498	0.873	0.205	0.907
		2/2		0.873	0.198	0.909
		2/3		0.862	0.168	0.905
		2/4		0.873	0.378	0.899
		2/5		0.872	0.365	0.9
		2/6		0.863	0.302	0.898
		3/1	0.498	0.86	0.272	0.895
		3/2		0.87	0.221	0.901
		3/3		0.862	0.168	0.905
		¾		0.885	0.633	0.902
		3/5		0.891	0.562	0.908
		3/6		0.869	0.448	0.904
		4/1	0.498	0.858	0.22	0.899
		4/2		0.86	0.19	0.901
		4/2		0.862	0.168	0.905
		4/2		0.863	0.459	0.902
		4/2		0.865	0.425	0.904
		4/2		0.866	0.395	0.909
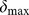	Dominant	1/1	0.104	0.04	0	0.038
		2/1	0.104	0.043	0	0.034
		2/2		0.042	0	0.034
		2/3		0.04	0	0.038
		2/4		0.042	0	0.032
		2/5		0.042	0	0.03
		2/6		0.04	0	0.037
		3/1	0.104	0.045	0	0.032
		3/2		0.046	0	0.033
		3/3		0.042	0	0.039
		¾		0.041	0.004	0.033
		3/5		0.044	0.002	0.037
		3/6		0.042	0.001	0.039
		4/1	0.104	0.042	0	0.039
		4/2		0.043	0	0.039
		4/3		0.042	0	0.039
		4/4		0.042	0.001	0.04
		4/5		0.043	0.001	0.039
		4/6		0.042	0	0.041

**Table 15:** Power analysis continues.

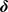	GM	MM/MR	S-TDT	TDT	rTDT	MI-TDT
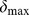	Additive	1/1	0.08	0.055	0	0.07
		2/1	0.08	0.054	0	0.061
		2/2		0.056	0	0.065
		2/3		0.055	0	0.07
		2/4		0.053	0.002	0.055
		2/5		0.055	0.002	0.058
		2/6		0.055	0.001	0.062
		3/1	0.08	0.052	0	0.055
		3/2		0.056	0	0.06
		3/3		0.055	0	0.07
		¾		0.056	0.007	0.059
		3/5		0.065	0.005	0.069
		3/6		0.059	0.003	0.068
		4/1	0.08	0.055	0	0.065
		4/2		0.055	0	0.066
		4/3		0.055	0	0.07
		4/4		0.057	0.003	0.07
		4/5		0.058	0.003	0.071
		4/6		0.058	0.002	0.075
	Recessive	1/1	0.077	0.066	0	0.084
		2/1	0.077	0.064	0	0.078
		2/2		0.066	0	0.081
		2/3		0.066	0	0.084
		2/4		0.063	0.002	0.07
		2/5		0.065	0.002	0.072
		2/6		0.065	0.001	0.075
		3/1	0.077	0.061	0.001	0.068
		3/2		0.065	0	0.075
		3/3		0.066	0	0.083
		¾		0.067	0.008	0.074
		3/5		0.078	0.006	0.083
		3/6		0.07	0.004	0.083
		4/1	0.077	0.064	0.001	0.077
		4/2		0.065	0	0.079
		4/2		0.066	0	0.083
		4/2		0.067	0.004	0.084
		4/2		0.068	0.003	0.086
		4/2		0.07	0.002	0.09

When the linkage disequilibrium was at its moderate level (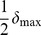), dominant models had the highest power following by additive and recessive models. While the power of MITDT-ONE ranged between 0.73 (0.94) and 0.84 (0.89), the power of rTDT ranged between 0.042 (0.17) and 0.45 (0.68) when 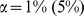. When linkage disequilibrium was at its maximum (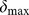), all testing procedures lacked power because the value of 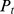 in (8) was close to 0.5 (this value was exactly 0.5 in validity analysis). When 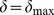, recessive models had the highest power, following by additive and dominant models, which was a reserve observation for 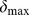. Over all, MITDT-ONE was the only method that provided the highest power at any significance level.

### Real Data: U.K. Warren Family

We illustrate the robustness of the MITDT-ONE for type 1 diabetes at insulin dependent diabetes mellitus 2 locus (IDDM2) on chromosome 11p15. At our request, Neil Walker of the Juvenile Diabetes Research Foundation/Wellcome Trust Diabetes and Inflammation Laboratory (JDRF/WT DIL) compiled data from 475 families with *two* affected offspring from the U.K. Warren Families for 52 SNPs. This data set was analyzed by [17] to demonstrate the method of MI-TDT for two affected offspring. The author of [21] used extensive logistic regression studies on the same data set, and identified −23 *Hph*I, +1,140A/C, +1428 *Fok*I, and VNTR as significant SNPs. The same SNPs as in [21] and six more were also identified by [17].

We considered the same U.K. Warren Families but chose the first affected child from each family to have only *one* affected offspring to demonstrate the performance of MITDT-ONE and rTDT. For the MITDT-ONE, we need to know frequencies of marker allele 1 (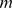) and disease allele (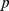) for each SNP. The values of 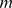 were provided to us along with the data set, except two (VNTR (DIL967) and TH micro' Z (DIL950)), but not the values of 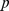. McGinnis (1998) [18] showed that TDT was able to detect linkage and its power exceeded 0.5 only when 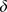 was close to 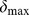 and allele frequencies 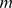 and 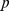 were similar in magnitude at the marker and disease locus. Therefore, we chose optimal values for 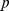 by assuming 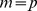.

The percentage of missing genotypes ranged from low (4% for DIL977) to high (52% for DIL997). Table 16 reports 18 significant SNPs out of 52 at 5% significance level for complete genotypes. Since we tested 52 SNPs, we applied Bonferroni multiple testing procedure at 0.05% significance level or 99.95% confidence level, and identified seven significant SNPs (underlined 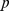-values). Since percentage of missing genotypes ranged from small to high, one should be cautious to declare significant SNPs when missing genotypes are ignored. Since DIL950 was insignificant for complete data, we dropped it from the computation of MITDT-ONE and rTDT. DIL967 was significant for complete data but its marker allele were not provided to us. Since we did not have any knowledge about the value of 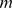, and did not want to assign any preferential value, we considered equal frequencies for 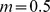 and 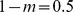.

**Table 16:** Type I Diabetes (IDDM): The significant SNPs for complete data.

SNP	Variant	%	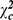	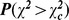
DIL997	C/T	52	4	0.0455003
DIL996	C/T	28	4.2631579	0.0389475
DIL989	C/T	26	4.7407407	0.0294564
DIL985	C/T	42	6.1084337	0.0134538
DIL984	G/A	22	3.8571429	0.0495346
DIL977	G/A	4	17.386831	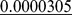
DIL976	T/G	36	10.940828	0.0009407
DIL975	C/T	30	11.571429	0.0006697
DIL974	A/C	30	16.568966	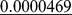
DIL973	T/C	16	14.069767	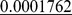
DIL971	G/C	20	10.971429	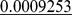
DIL969	A/T	6	23.027397	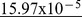
DIL967	VNTR	6	21.300341	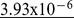
DIL965	T/C	20	14.901478	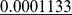
DIL963	A/C	22	10.414286	0.0012504
DIL954	C/T	36	6.2857143	0.0121715
DIL3872	C/G	18	7.4745763	0.0062576
DIL2048	C/T	12	3.7815126	0.0518218

The third, fourth, and fifth columns show the percentages of missing data, the TDT statistics for complete data, and uncorrected p-values at 5% significance level. The significance SNPs are shown by underlined 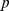-values for Bonferroni at 0.05% significance level.

The MITDT-ONE and rTDT could verify if the significant SNPs for complete data are also significant when missing genotypes are taken into account. However, if either method could not reach significant result as in complete case, it does not mean that these SNPs are insignificant. It simply means that both methods reach an inconclusive decision. Moreover, the number of significant SNPs could be smaller when either test is employed, compared to the number of significant SNPs for complete data. Out of 18 significant SNPs in complete cases, MITDT-ONE (rTDT) verified seven (three) to be significant (Table 17). The MITDT-ONE as well as rTDT found 23 *Hph*I, +1428 *Fok*I, and VNTR as significant SNPs as in [21] and [17]. Furthermore, MITDT-ONE identified four more same SNPs in [17] as significant; hence, we suggest researchers to investigate these SNPs as possible casual variant genes.

**Table 17:** The Significant SNPs at 5% significance level when the MITDT-ONE is applied.

SNP	Variant	Name	dbSNP	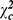	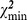	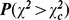	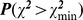
DIL977*	G/A	+1,428 *Fok*I	rs3842756	17.39	16.82	0.0000305	0.0000412
DIL973	T/C	+1,127 *Pst*I	rs3842752	14.07	8.00	0.0001762	0.0046696
DIL971	G/C	+805 *Dra*III	rs3842748	10.98	6.13	0.0009253	0.0133311
DIL969*	A/T	−23 *Hph*I	rs689	23.03	21.44	15.97× 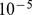	36.48×10^−5^
DIL967*	VNTR	VNTR	-	21.30	18.27	3.93× 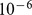	0.0000192
DIL965	T/C	−2,221 *Msp*I	rs3842729	14.90	7.97	0.0001133	0.0047659
DIL963	A/C	−2,733A/C	rs3842727	10.41	4.68	0.0012504	0.0306104

The third and fourth columns show the name of the SNP defined in Barratt et al. (2004) and the SNP database, respectively. The fifth and sixth columns show the statistics for complete and incomplete data when MITDT-ONE is applied, respectively. The seventh and eight columns show the type I errors of the columns fifth and sixth, respectively.

* are SNPs found in association by using rTDT.

## Discussion

Sebastiani et al. [16] proposed to handle missing genotypes of parents or offspring in a nuclear family with one affected offspring. However, rTDT produces a conservative test and lacks power. Hence, we proposed MITDT-ONE to correct the problems of rTDT. The MITDT-ONE takes population frequencies of marker allele 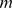 and disease allele 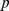 into account in the rTDT method. With these 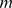 and 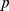 values, we restrict the domain of rTDT to have much better estimates for the maximum values of 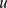 and 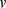.

The minimum values of the interval estimates of MITDT-ONE and rTDT make a decision against the null hypothesis of no linkage. One of the advantages of using MITDT-ONE is that significance results achieved by complete data is ratified when the minimum value of the interval estimate is smaller than the value of TDT for complete data. The other advantage of our method is that it allows researchers to implement our method to any missing rates. As discussed in the introduction, many studies deal with missing genotypes in parents but not in offspring. Moreover, these methods assume some missing mechanism (e.g., MAR) to recover parental genotypes. Thus, another strength of MITDT-ONE is that it does not assume any missing model but simply considers the Mendelian Inheritance property to define all possible admissible genotypes in parents or offspring. Also, MITDT-ONE and rTDT become classical TDT when 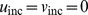.

In the construction of MITDT-ONE, we consider cases where all genotypes of family members are missing (Case 1). It is intuitive that since these families do not have any information they should be ignored from the study. We suggest that these families be omitted from the data if only one SNP is studied. However, if more than one SNP are studied then we suggest keeping them in the computation of MITDT-ONE to have same number of families for each SNP.

In summary, simulation studies show that MITDT-ONE controls type I error rates very well and produces high power when degree of linkage disequilibrium is mild.

*More than one offspring:* rTDT for two affected offspring was proposed by [17]. However, it was a conservative test and had low power. Hence, Alpargu [17] proposed MI-TDT to remedy the problems. With the motivation of Alpargu [17], we proposed MITDT-ONE. Both MITDT-ONE and MI-TDT correct the problems arising from rTDT. Theoretically, it is possible to propose our method for families with at least three and more affected offspring. However, the computation will be tedious because the number of missing cases increases as the number of affected offspring increases. Moreover, in the linkage studies it is very rare to have more than two affected offspring.

*Multiple alleles:* We proposed MITDT-ONE for bi-allelic cases. However, it is possible to extend to multi-allelic cases. We consider two approaches that have been used in practice [22], [23]. In the first approach, all alleles except the allele of interest are grouped as allele 2, and the MITDT-ONE for bi-allelic case is applied [22]. In the second approach, if we have 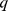 alleles, then for each allele, the first approach is applied to obtain 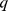 MITDT-ONE statistics, then the largest MITDT-ONE is chosen as the test statistic [23] to make a decision about significant gene.
